# Experiences and perceptions of palliative care patients receiving virtual reality therapy: a meta-synthesis of qualitative studies

**DOI:** 10.1186/s12904-024-01520-5

**Published:** 2024-07-23

**Authors:** Yufei Huang, Cunqing Deng, Meifang Peng, Yanping Hao

**Affiliations:** 1 College of Nursing, Guangzhou Medical University, Guangzhou, Guangdong China; 2 Department of Internal Medicine, Affiliated Cancer Hospital and Institute, Guangzhou Medical University, Guangzhou, Guangdong China

**Keywords:** Virtual reality, Palliative care, Experience, Perception, Perspective, Systematic review, Qualitative

## Abstract

**Background:**

The combination of virtual reality (VR) and palliative care potentially represents a new opportunity for palliative care. Many previous studies have evaluated the application of VR therapy to patients with advanced disease receiving palliative care. However, patient-perspective reviews to comprehensively understand the actual experiences and feelings of patients and provide practical guidance for designing future studies are currently lacking. This review of qualitative evidence aimed to explore the experiences and perceptions of patients receiving VR therapy in palliative care.

**Methods:**

This study was conducted in accordance with the Enhancing Transparency in Reporting the Synthesis of Qualitative Research (ENTREQ) statement guidelines. Ten databases, namely, PubMed, Web of Science, EBSCO, OVID MEDLINE, Scopus, John Wiley, ProQuest, CNKI, WANFANG DATA, and SinoMed, were searched, and qualitative and mixed studies from the establishment of each database to June 30, 2023 were included. The Joanna Briggs Institute Critical Appraisal Checklist for Qualitative Research was used to assess the quality of the included studies. The data included in the literature were analyzed and integrated by “thematic synthesis” to formalize the identification and development of themes.

**Results:**

The nine selected studies altogether included 156 participants from seven hospice care facilities of different types and two oncology centers. Three key themes were identified: experiences of palliative care patients in VR therapy, the perceived value that palliative care patients gain in VR therapy, and perspectives of palliative care patients toward using VR therapy.

**Conclusions:**

The patients’ feedback covered discomfort caused by VR devices, good sense of experiences, and situations that affected the interactive experience. Some patients were unable to tolerate VR therapy or reported newer forms of discomfort. The findings indicated that VR therapy may be an effective approach to relieve patients’ physical and psychological pain and help them gain self-awareness. Moreover, patients showed a preference for personalized VR therapy.

**Supplementary Information:**

The online version contains supplementary material available at 10.1186/s12904-024-01520-5.

## Background

With the aging of the global population and the high incidence of cancer, the demand for palliative care is increasing rapidly [1, 2]. Over the past few decades, palliative care has evolved from a nursing philosophy focused on end-of-life care to a specialized profession with the provision for comprehensive supportive care throughout the disease trajectory for patients with advanced disease [3]. A patient’s palliative care needs can be classified as physical, emotional, spiritual, social, and informational, which are often closely associated with each other [3].

However, patients who require palliative care, such as those with chronic, incurable, progressive, and unpredictable clinical conditions, often develop severe physical and psychological symptoms, such as dyspnea, fatigue, exhaustion, and anxiety, as a result of treatment and disease progression [4–6]. Moreover, these patients are often confined to their beds or rooms due to their deteriorating health [7]. Thus, they are deprived of opportunities to socialize and engage in leisure activities. These issues can affect the quality of the patients’ remaining lives [5, 8]. Engaging in meaningful activities is one way to improve patients’ quality of life and support them to live with dignity [7], and the combination of palliative care and Virtual reality can give them a new opportunity in this regard.

Virtual reality (VR) is a combination of immersive experiences, stereovision, and motion-capture technology in which real-world visual perception is used in a fully artificial computer-generated environment [9]. VR experience is usually achieved with headsets that can locate the user's movements. In general, these VR headsets consist of a screen housed in a frame (or headset) strapped or fitted to the user’s head, and there is a pair of lenses fixed between the panels and eyes [10]. Users will have a controller in each hand, or be delivered virtual representations of hands, to control aspects of the experience. VR technology can control the visual and auditory scenes of the participants, and this control can provide a softer or amplified version of the virtual experience of reality [11]. With advancements in VR technology, the convenience and accessibility of VR have allowed its gradual application to the fields of medicine and psychology [11–16]. Depending on the actions within VR, VR therapy can be divided into passive VR therapy and active VR therapy. The passive VR therapy is when participants are immersed in the VR environment, such as natural scenery, but they cannot interact with the VR environment or object[13]. In active VR therapy, patients can interact in VR based on pre-designed scenarios or games[14]. VR-based therapy has been shown to be an effective and accessible treatment for acute and chronic pain [14, 15, 17]. Multiple studies have shown that VR Intervention leads to significant decreases in anxiety and depression levels in patients, its positive effects on subjective well-being have also been confirmed [18–20]. In comparison with the corresponding findings before VR treatment, patients with advanced cancer experienced a statistically significant reduction in symptoms such as pain, fatigue, drowsiness, and shortness of breath after VR therapy [21, 22]. A recent hybrid study [23] found that the total Edmonton Symptom Assessment Scale (ESAS) score was significantly lower in patients receiving palliative care after VR treatment, suggesting that VR treatment is effective in reducing the symptom burden of patients receiving palliative care. VR-based therapy appears to be beneficial for patients.

User experience (UX), which includes immersive UX, affective UX, lived UX, and interactivity UX, represents a pure subjective feeling established by users in the process of using a product [24]. The perceived value is the user's psychological assessment of a product or service after measuring their perception, which is the trade-off between earned benefits and sacrifices [25].

Many previous studies have evaluated the application of VR to patients with advanced disease and palliative care. These studies focused primarily on assessing the effectiveness of symptom management in patients or the feasibility of interventions [21, 22, 26–28]. Some studies have also used questionnaires to understand patient satisfaction and opinions after the end of the intervention [21, 29, 30]. However, to the best of our knowledge, none of the previous reviews have focused on patients’ experience of VR therapy in palliative care. Most of the existing reviews have comprehensively affirmed the effectiveness of VR therapy from quantitative data [12, 31] or a holistic view of the use of VR in palliative care [32]**.** However, patient-perspective reviews to comprehensively understand the actual experiences and feelings of patients and provide practical guidance for designing future studies are currently lacking.

Thus, to understand the real value of VR therapy for patients and the problems encountered in the process, we integrated the experiences and perceptions of palliative care patients receiving VR therapy. We expect that the findings will highlight the directions for better implementation of VR capabilities in palliative care in the future.

## Methods

We registered the qualitative review protocol in PROSPERO (CRD42023453177). The systematic review was conducted in accordance with the Enhancing Transparency in Reporting the Synthesis of Qualitative Research (ENTREQ) statement guidelines [33].

### Search strategy

Ten databases, namely, PubMed, Web of Science, EBSCO, OVID MEDLINE, Scopus, John Wiley, ProQuest, China National Knowledge Infrastructure (CNKI), WANFANG DATA, and Chinese biomedical literature service system (SinoMed), were searched for this review. We reviewed the search terms used in other systematic reviews in this area, and identified “palliative care,” “end-of-life,” “end of stage,” “terminal,” “hospice,” “dying,” “Virtual Reality,” “VR,” “Virtual technology,” and “virtual reality goggle” as screening keywords. Qualitative and mixed studies from the establishment of each database to June 30, 2023 were included in the searches. The full database search strategy is provided [see Additional file 1]. We also performed a lateral search of citations of the included articles and relevant systematic reviews.

Qualitative studies were defined as those using methodologies such as phenomenology, ethnography, grounded theory, hermeneutics, and narrative or thematic analysis, and/or primarily analyzing textual rather than numerical data [34]. Mixed methods research is a type of research in which a researcher or team of researchers combines elements of qualitative and quantitative research approaches [35]. We only included mixed studies that fully elaborated the qualitative study.

Studies were included when they met the following criteria: (1) The study population consisted of palliative care patients and the patients received any type of VR-related therapy. (2) The research method was a qualitative study or a mixed methods study including qualitative data. If the study explored the experiences and perceptions of multiple people, the data of the patients were included. (3) The study was written in English or Chinese. We excluded studies in which patients did not actually use VR services, articles without the full text available, and articles with incomplete data.

### Study selection and data extraction

Search results were imported into NoteExpress and duplicates were removed. Study selection was independently completed by two researchers (H.-Y.F. and D.-C.Q.) and cross-checked according to the inclusion and exclusion criteria. Disagreements were resolved by discussion and evaluation by a third researcher (L.-H.Y.) when required. Article selection involved two steps: (1) initial assessment of the title and abstract and exclusion of the irrelevant literature; (2) further assessment of the full text and final evaluation of the suitability of the study. We extract only the necessary basic information about the article as well as the qualitative findings. Excel 2021 and a self-made extraction table were used for data extraction. The extracted data mainly included information regarding the (1) author; (2) year of publication; (3) country; (4) design; (5) participants; (6) aims of the study; (7) phenomenon of interest; and (8) themes/results.

The two researchers then independently evaluated the literature quality using The Joanna Briggs Institute Critical Appraisal Checklist for Qualitative Research to evaluate the quality of the included studies [36]. A total of 10 items were evaluated, each of which was categorized as “Yes/No/Unclear/Not applicable.” Disagreements in these evaluations were also resolved by discussion and additional evaluation when required.

### Data synthesis

The data included in the literature were analyzed and integrated by “thematic synthesis” to formalize the identification and development of themes. Thematic synthesis, which was summarized and presented by James Thomas and Angela Harden in 2008, is a method to synthesize the research results by forming themes [37]. The explicit recording of the development of themes is a central aspect of this method [37]. This process was performed over three stages that overlapped to some degree: (1) In stages 1 and 2, two authors (H.-Y.F. and D.-C.Q.) coded all results and findings into *Nvivo* 20 on the basis of their meaning and developed descriptive themes. (2) In stage 3, the two researchers used the descriptive themes to generate a set of results and analytical themes. Finally, the analytical themes were refined again to form the key themes. The two researchers independently conducted data analysis and integration in each stage, and a third researcher (L.-H.Y.) participated in the discussion in case of any disagreement.

## Results

### Search results

**Fig. 1:**
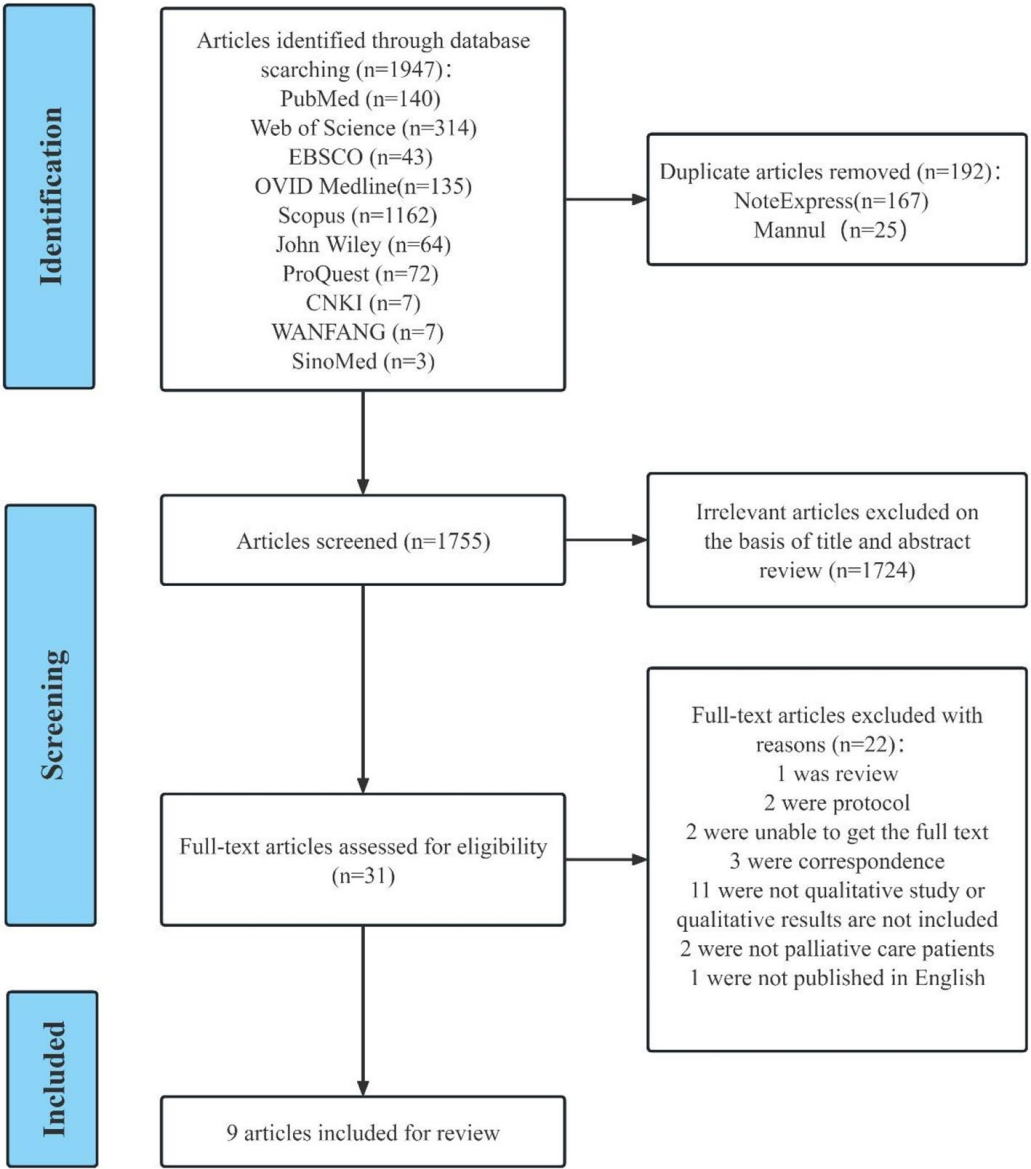
Flowchart of the search and selection process

A total of 1947 articles were obtained after searching the databases and tracking the references of the included studies (Fig. 1). After excluding duplicates and screening the titles and abstracts, the full texts of 31 studies were reviewed. Finally, nine studies fulfilled the eligibility criteria and were included in our systematic synthesis.able.

### Study characteristics

The nine studies included one study conducted in Australia [30], two in the United Kingdom [38, 39], five in the United States [40–44], and one in Canada [45]. Six studies used mixed research methods [30, 38, 40, 42–44]; two were qualitative studies [39, 41]; and one was a case report [45]. Almost all studies collected data through semi-structured interviews [30, 38–41, 44], while three studies used a previously designed questionnaire containing qualitative questions [42, 43, 45]. The nine studies altogether included 156 participants from seven hospice care facilities of different types and two oncology centers. A summary of the study characteristics is shown in Table 1, and a summary of study findings is shown in Table 2.

**Table 1:** Characteristics of the included studies

Study information	Aims	Study design	Participant details	Settings
Austin et al. Australia, 2022 [30]	To determine the feasibility and preliminary effectiveness for larger randomized controlled trials of 3D-head mounted VR for managing cancer pain in adults	Mixed-method research; semi-structured interview; thematic analysis	13 people with cancer pain receiving palliative care	Hospital and community care
Kelleher et al. United States, 2022 [40]	To examine the feasibility, acceptability, safety, and impact of a 30-min virtual underwater/sea environment (VR Blue) for reducing pain and pain-related symptoms in patients with advanced colorectal cancer	Mixed-method research; semi-structured interview; thematic analysis	20 participants with stage IV colorectal cancer and moderate-to-severe pain	The Duke Cancer Institute
O'Gara et al. United Kingdom, 2022 [38]	To codesign and test a VR intervention incorporating relaxation and compassionate mind training, determine its acceptability/feasibility in an oncology setting, and evaluate its impact on physical/psychological well-being and quality of life	Mixed-method research; semi-structured telephone interview; framework analysis	21 participants undergoing cancer treatment, recovery, or palliative care	A specialist cancer center
Ryu et al. United States, 2022 [41]	To assess the feasibility of delivering an avatar-facilitated life review intervention for patients with cancer	Qualitative research; semi-structured interview; framework analysis	12 patients receiving outpatient palliative care	Outpatient palliative care
Brungardt et al. United States, 2021 [42]	To evaluate the implementation measures of feasibility, usability, and acceptability of a VR-based music therapy (MT) intervention	Mixed-method research; structured interview; inductive and deductive approaches	33 hospitalized patients receiving palliative care	Hospitalized palliative care
Lloyd et al. United Kingdom, 2021 [39]	To explore the acceptability and potential benefits of immersive VR for people with life-limiting conditions	Qualitative research; semi-structured interview; thematic analysis	19 hospice patients	Inpatients in a hospice setting
Johnson et al. United States, 2020 [43]	To examine the utility of VR for palliative care patients	Mixed-method research; structured interview; thematic analysis	12 adult patients diagnosed with life-limiting illness	A free-standing hospice facility
Ferguson et al. United States, 2020 [44]	To explore the acceptability, tolerability, and subjective experience of VR as therapeutic recreation for hospice patients living with dementia	Mixed-method research; semi-structured interview; content analysis	25 hospice patients living with dementia cared for by a local hospice agency	Community Hospice Agency
Weingarten et al. Canada, 2020 [45]	To share a girl’s experience of using VR	Case report; structured interview; experience sharing	A 12-year-old girl with high-risk acute myelocytic leukemia	/

**Table 2:** Summary of the findings of the included studies

**Study**	**Intervention** (length, content, form)	**Phenomenon of interest**	**Themes/Results**
Austin et al. Australia, 2022 [30]	**Length:** the length of the video. **Content:** viewed a 3D VR experience called Nature Trek®. **Form:** non-interactive; standardized form	Experiences and challenges of using the VR platforms	Four topics and nine subtopics: (1) Usability: 1) application, 2) learning, 3) satisfaction; (2) Presence: 1) spatial awareness, 2) engagement, 3) realism; (3) Cyber sickness: 1) physical effects; (4) Expectations: 1) future use, 2) other uses
Kelleher et al. United States, 2022 [40]	**Length:** 30 min. **Content:** virtual underwater/sea environment (VR Blue). **Form:** non-interactive; standardized form	Preferences, thoughts, and feelings about the VR experience	Five themes: (1) use of VR technology; (2) timing of VR Blue session; (3) enjoyment of VR Blue session;(4) VR Blue graphics;(5) areas for improvement and next steps
O'Gara et al. United Kingdom, 2022 [38]	**Length:** all sessions approximately 10 min long. **Content:** Choice of male or female voice, choice of a VR beach, mountain or forest scene. VR1: Adapting to wearing VR headset and being in a VR environment;VR2: Simple soothing/ breathing exercise, introduction to compassionate mind training (CMT). VR3: Simple CMT exercise. **Form:** non-interactive; semi-structured format	Feedback for or experience of the intervention	Three themes: (1) practical issues;(2) immersion; (3) impact of intervention
Ryu et al. United States, 2021	**Length: **30-40 min. **Content:** Patients began at the “child” avatar stage, progressing to “teenager”, “adult”, and ending in the “elder” stage. Life review questions and prompts began by situating the virtual body in the chosen virtual environment and were adapted to the selected developmental stage. Patients’ avatar narratives were screen recorded, and processed as movie files to create a legacy document. **Form:** interactive; semi-structured format	Impact on quality of life, satisfaction with the VoicingHan experience, and recommendations for improving the experience	Three Phases, five categories, and thirteen themes: Phase 1: Construction of individual performance. Phase 2: Life review and narrative reconstruction (1) Psychosocial development and life review: 1) childhood lost and rediscovered, 2) new 3) meaning to old actions, 4) seeing the emergent self, 5) carving identity from adversity, 6) following the heart, 7) finding oneself after heartbreak, 8) affirming life choices and circumstances, 9) struggling to see the future; (2) Reconstructed meaning: 1) agency, 2) retrospective sense-making and social reconstruction; Phase 3: Instrumental and psychological influences:(1) instrumental satisfaction, (2) psychological connection
Brungardt et al United States, 2021 [42]	**Length:** two days. **Content:** VR-based music therapy (MT) intervention. Video content allowed participants to be immersed in a nonanimated nature environment including natural sounds. **Form:** non-interactive; semi-structured format	Experiences of VR-MT and feedback for iterative design	Three categories and nine themes: (1) Acceptability: 1) positive anticipation about VR-MT, 2) appreciation of ability to create a customized experience, 3) variation in physical acceptability of the VR headset and headphones; (2) User experience: 1) emotional response to VR-MT, 2) respite from medical circumstances, 3) physical response to VR-MT; (3) Inputs on intervention refinement: 1) VR-MT length and frequency of use (dosing), 2) VR environments, 3) time of delivery
Lloyd et al. United States, 2021 [39]	**Length:** 30 min. **Content:** The VR session began with the facilitator (BA) demonstrating the technology when the participant was immersed in a simulated underwater environment. They were then asked to decide upon a destination of their choice. **Form:** non-interactive; semi-structured format	Experiences of and responses to the VR session	Three themes: (1) the capacity for new experiences; (2) the capacity to reconnect with the past; (3) the capacity to forget
Johnson et al. United States, 2020 [43]	**Length:** 30 min. **Content:** 9 applications include auditory and visual components: 360 Photos, Meditation, Apollo 11, Bait!, Feel: The Must of the Sea, Bear Island, the Blu, Coaster, and Hello Mars. Participants were free to switch between VR applications and terminate the intervention at any time if desired. **Form:** interactive (little or nothing); semi-structured format	Perceptions of the VR experience	Four themes: (1) found the VR technology easy to use; (2) experienced no difficulties in operation; (3) would recommend VR to a friend in a similar situation; (4) experienced no adverse effects
Ferguson et al. United States, 2020 [44]	**Length:** 30 min. **Content:** The VR experience was a short YouTube VR 360 beach scene video, a 3.5-min video looped for up to 12 times. **Form:** non-interactive; standardized form	Perceived experience of VR	Four themes: (1) narration; (2) affirmation; (3) comfort level; (4) lack of fulfillment
Weingarten et al. Canada, 2020 [45]	**Length:** 5-10 min. **Content:** Watched Customized 360° videos in VR, including figure skating, flying, roller coaster rides, kayaking, and more. **Form:** non-interactive; personalized format	Experience of VR	Three sections: (1) general questions regarding LL's condition before the session; (2) logistics of the session itself; (3) LL's response to the session

### Quality assessment

The results of the literature quality evaluation of the included studies are provided in Table 3. All 10 items were rated “YES” in only one study [39]. Nine, eight, and seven items were rated “YES” in two studies [41, 42], four studies [38, 40, 43, 44], and two studies [30, 45], respectively. None of the studies were excluded based on qualitative appraisal. Most studies lacked information for the item assessing the cultural and theoretical background of the researchers [30, 38, 40, 41, 43–45]. More than half of the included studies did not clearly report the impact of the researchers on the research [30, 38, 40, 42, 44]. In addition, two studies showed no congruity between the research methodologies and their representation and analysis of data [30, 43].

**Table 3:** Joanna Briggs Institute critical appraisal of the included studies

Study	I1	I2	I3	I4	I5	I6	I7	I8	I9	I10
Austin et al. [30]	Y	Y	Y	N	Y	U	U	Y	Y	Y
Kelleher et al. [40]	Y	Y	Y	Y	Y	U	U	Y	Y	Y
O'Gara et al. [38]	Y	Y	Y	Y	Y	U	U	Y	Y	Y
Ryu and Price [41]	Y	Y	Y	Y	Y	U	Y	Y	Y	Y
Brungardt et al. [42]	Y	Y	Y	Y	Y	Y	U	Y	Y	Y
Lloyd and Haraldsdottir [39]	Y	Y	Y	Y	Y	Y	Y	Y	Y	Y
Johnson et al. [43]	Y	Y	Y	N	Y	U	Y	N	Y	Y
Ferguson et al. [44]	Y	Y	Y	Y	Y	U	U	Y	Y	Y
Weingarten et al. [45]	Y	Y	Y	U	Y	U	Y	Y	U	Y

*Y* yes, *N* no, *U* unclear

The average consistency rate of the two researchers after independent quality assessment was 0.819, indicating good consistency. An additional file shows the results of each quality assessment item of the nine articles rated by the two researchers [see Additional file 2].

### Results of the synthesis analyses

We also identified three key themes and seven analytical themes. The three key themes included (1) experiences of palliative care patients in VR therapy, (2) perceived value that palliative care patients gain in VR therapy, and (3) perspectives of palliative care patients toward using VR therapy. An additional file shows the thematic synthesis process and proofs [see Additional file 3].

#### Theme 1: Experiences of palliative care patients in VR therapy

The experience of using the VR hardware device was affected by the weight of the device, the manner in which it was to be worn, the difficulty in using it, and the physical condition of the patient[30, 40–44]. There were mixed views about the participants’ experiences of the VR therapy process [30, 39, 41, 44]. On the other hand, the experience of the VR interactive process was influenced by the realism of the virtual scene, the completeness of the interventional content, and whether the environment was quiet[30, 38, 41, 42, 44].

#### Analytical theme 1: The experience of using VR hardware devices

In terms of hardware, participants reported difficulty wearing VR-related devices comfortably, and experienced sore shoulders due to repeated adjustments to the equipment [42–44]. Moreover, the weight of the VR device itself also bothered the participants, mainly because of the headphones and facial devices [40, 44].

*Took headset off saying it was too heavy—PI asked about any pain from it being heavy—they asked for them to be put on again, but again quickly removed after looking around and saying they were too heavy.* < *Ferguson, 2020* > [44]

In terms of operation, some participants found the VR device was easy or not very difficult to use initially [40, 43]. However, some participants reported difficulty in learning the button configuration of the remote controller [43]. Nevertheless, some participants could move around the VR environment easily after a few minutes of practice or getting some guidelines [30, 41].

*Kaitlyn who was having trouble connecting with her avatar at first was asked to pretend she was doing sports (“swing a tennis racquet”; “pretend you’re swimming”) which effectively synched her own motion with the motion she saw performed back; this relaxed her and helped her engage in conversation.* < *Ryu, 2022* > [41]

#### Analytical theme 2: The experience of the VR therapy process

For most participants, VR therapy was indeed a different experience [39, 44]. Most participants reported a good sense of immersion [30, 39, 41, 44], and participants could see a series of “very realistic” virtual scenes such as “the ocean,” “the sun,” and “the fish swimming.” When they were in the virtual environment, participants thought they had a good interactive experience with the virtual environments or their own virtualized forms [41].However, some participants also reported the opposite experience [30, 41, 42]. One of the reasons for poor human–computer interaction experiences was monotonous VR intervention content [42]. Moreover, noise from the outside environment and the intrusion of unrelated people also disrupted the experience of the participants [30, 41]. The presence of other family members appears to affect the patient's immersion in VR therapy [41].

“*I found it very distressful and distracting, when she walked in just then… who was that anyhow… I was in my place and that just blew it all out… if anyone walked in, it would be the same. OK, I’m not ready anymore but let’s go on.”* < *Ryu, 2022* > [41]

During the process of using VR therapy, due to the peculiarities of VR technology, the participants experienced some slight discomfort [30, 42], such as nausea, dizziness, claustrophobia, etc. The physical condition of the patients seemed to affect the degree of completion of the VR experience. Some patients failed to obtain a complete experience due to the poor functional status caused by the disease [38, 44].

*“I find breathing exercises really frustrating … I have tumours in my lungs, the amount I can inhale, the amount of time I can hold for is less than for other people. So, someone will say hold it this many beeps and then you can’t … you feel like you failed at it and you check out…”* < *O'Gara,2022* > [38]

#### Theme 2: The perceived value that palliative care patients gain in VR therapy

The patients’ gained perceived value after experiencing VR therapy can be summarized into two aspects: the actual effects of VR therapy and the value of the self-awareness provided by VR therapy. The former mainly included relief from physical pain [39, 42], and positive psychological and emotional support [30, 38, 39, 42, 44, 45], while the latter was an abstract concept expressed by the participants[39, 41, 42, 45].

#### Analytical theme 1: The actual effects of VR therapy in palliative care

On one hand, VR therapy is considered to help patients reduce or forget pain [39, 42]. On the other hand, participants thought that VR allowed them to separate themselves from the depressing situation or the frustration caused by their condition [42, 45].

*“It gave us something to do while not being able to leave the room, it was a new experience that was exciting and helped to distract from the depressing situation of being isolated from everyone.”* < *Weingarten, 2020* > [45]

A significant number of participants described feelings related to relaxation and calm after the VR experience [30, 38, 42, 44], such as “so relaxed and comfortable,” and reported feeling that they had entered another world. Moreover, the participants further gained happiness during the process, with more than one person mentioning “happiness,” and some people smiling happily in the process [39, 44].

*“A sense of euphoria. A sense of peace. A sense of calming that was not there before.” (62-year-old man)* < *Brungardt, 2021* > [42]

#### Analytical theme 2: The value of the self-awareness provided by VR therapy

In addition to these intuitive feelings, VR therapy also seemed to provide some symbolic value to the participants. For example, it awakened some precious memories [39], such as seeing the mountains that the participants had climbed in the past, “returning” to the place where they lived in their childhood, recalling the intimacy with their family, or other warm and beautiful experiences.

*“It brought back memories of generally we would go there on the Sunday. We lived about thirty-five miles away, so our Sunday outing would have been there. And my Mum and Dad would have been asking me to get out of the car, and I was only a small child at the time, but all I wanted to do was read my book, lie on a rug and read my book.”* < *Lloyd, 2021* > [39]

Besides, participants seemed to be able to re-understand their past behaviors and give them new meaning through VR [41]. In the virtual world, some people made the opposite choice to reality and created a new self [41], while others discovered a part of themselves that they had not paid attention to in the past, gaining a new perspective.

A significant proportion of patients reported that they could use VR to take a break from the constraints of reality, fulfill their wishes, and regain a sense of control over their lives [39, 42]. These wishes were originally unfulfilled due to their illness, but VR allowed them to change this. Participants reported that they could go to a place they wanted to go with the help of VR, or make up for their inner regrets. For example, a participant “went to New York” [39]. At the end of the VR therapy, the participants thought that life was something worth living [39, 45].

*“I can just put on headphones and kind of space out for a while and be in my own little world that I get to pick out. To a place you want to be. Not somewhere you have to be or stuck. Which is a piece of control you can take back a little bit.” (24-year-old woman)* < *Brungardt, 2021* > [42]

#### Theme 3: Perspectives of palliative care patients toward using VR therapy

On the basis of the participants’ responses, we inferred that the participants’ attitudes and preferences toward using VR were inconsistent, with some giving positive feedback [38–40, 42, 43]and others showing a lack of interest[38, 42–44].

#### Analytical theme 1: Attitudes before using VR therapy

One participant expressed concern before VR therapy because of her past claustrophobic experiences, and she feared that the closed visual device would cause her to experience fear again [42]. However, some participants showed good pre-acceptance of VR [38, 42]. For these people, the degree of pre-acceptance and engagement with VR therapy appeared to be related to past experiences with non-medical support measures [38, 42]. For example, participants who had been exposed to yoga or listened to music a lot were less likely to feel nervous before using VR.

*“It was really easy for me to pick out a playlist. I automatically started thinking about songs that I liked. So, it was really easy for me to think about if I wanted to space out, what I wanted to feel and listen to. The only hard part about it was just wondering what it was gonna be like.” (24-year-old woman)* < *Brungardt, 2021* > [42]

#### Analytical theme 2: Attitudes after using VR therapy

At the end of the VR therapy, the participants said they did not want to leave the wonderful virtual world built by VR [39, 44].

*“I’m sleepy, but I don’t want to leave this wonderful place”* < *Ferguson, 2020* > [44]

Some participants did not find VR therapy attractive even if they agreed that the scenery in VR was beautiful [44]. A participant did not like the other content (For example, compassion mind therapy) united at the time of VR therapy [38]. The feeling of fulfillment provided by VR therapy can persist to the next day [39]. Many patients thought that the effect of a single session of VR therapy was limited but the positive effects of the therapy were undeniable [38, 43].

*“I don’t think it will have a lasting impact…It definitely made the rest of the day easier … But the next day, the day after, I didn’t still have that same sense of calm, it was just kind of immediately after…”* < *O'Gara, 2022* > [38]

The majority of participants seemed optimistic about the potential for VR therapy in the palliative care setting and responded that they would recommend it to friends [43]. Most participants stated they would use VR in the future if it could really control pain and provide sufficient relaxation [30], but some participants responded that they did not need more VR therapy [44].

#### Analytical theme 3: Preferences for the VR therapy intervention

Participants presented a range of preferences related to the setup of VR interventions. First, some participants thought that VR therapy should be conducted in the evening because there was more free time in the evening [42]. Participants who preferred to undergo VR therapy early in the morning reported that it was the best time [42].

*“I don’t know the optimal time. Early mornings, I love. But it seems like that’s when I’m most emotional and upset is really the morning.” (69-year-old woman)* < *Brungardt, 2021* > [42]

Second, many participants preferred VR therapy conducted concurrently with cancer treatment; others preferred initiation of VR therapy on diagnosis; while some others preferred the use of VR therapy during periods of severe pain or negative emotions [40]. With regard to the frequency of intervention, some participants wanted to use VR therapy every day, while some thought that therapy performed multiple times a week was appropriate [40]. One participant responded that the frequency of therapy should be based on the patient’s preference [42].

*“You could do several different lengths. You could do a 10 min one. Some people would like an hour.” (47-year-old woman)* < *Brungardt, 2021* > [42]

Finally, while most participants reported that their close ones would be interested and willing to participate in the use of VR [40], participants seemed to prefer using VR independently [45].

## Discussion

### Principal findings

We used a thematic analysis approach to review nine studies that explored the experiences and perceptions related to the use of VR therapy among palliative care patients. The three key themes were the experiences of palliative care patients in VR therapy, the perceived value that palliative care patients gain in VR therapy, and the perspectives of palliative care patients toward using VR therapy.

Overall, participants reported that the VR therapy experience was not bad. They believed that VR therapy could help relieve some forms of physical and psychological discomfort. VR therapy offered some symbolic value for patients receiving palliative care, such as evoking fond memories, reflecting on past actions and events, reshaping and discovering new selves, and implementing wishes that remained unfulfilled due to the illness. Most patients were willing to use VR therapy again in palliative care. However, the findings also revealed some problems and obstacles, such as poor experiences due to the devices or the VR therapy content, and participants reported a variety of different preferences related to aspects such as the timing, frequency, and length of the interventions. Apparently, palliative care patients were more interested in personalized VR therapy.

#### Preliminary preparation for VR therapy: device, content, environment

Remarks on the size of the VR device and the discomfort of wearing it were not unique [46–48]. The design of VR glasses/headsets is challenging because of the trade-off between appearance and performance requirements [49, 50]. The weight, weight distribution, and wearing style of different VR devices exert different degrees of pressure on the weight-bearing parts of the face, affecting the overall comfort of the user [51]. A lighter weight can reduce user discomfort, and according to the concepts of ergonomics, the maximum mass of the head-mounted display should not exceed 1000 g [52]. The prolonged static posture or repetitive arm postures during VR interactions can further aggravate the discomfort and fatigue [49]. Moreover, since patients in palliative care may have worse-than-normal levels of physical activity, VR therapy protocols that necessitate periods of continuous standing or sitting can magnify the burden of the weight of the device and reduce patient comfort [53]. Therefore, these patients should be provided with supporting devices to reduce the weight and fatigue caused by the VR devices during VR therapy. Another important aspect is that VR headsets generate a lot of heat during operation, and this heat can also affect the user’s experience and comfort. Wang et al. [54] found that the overall subjective thermal discomfort associated with the use of VR headsets increased with the time of use. Thus, the design of VR headsets should consider reducing the display coverage area of the user's face, especially in areas with high sweating rates [54].

The participants’ experience was also affected by the monotonous interactive content. Interactivity with the VR environment can help keep participants connected to the live experience, optimizing the user’s presence and enhancing the sense of immersion to some extent [24]. However, too much interaction can make motion sickness more intense. The frequency or severity of motion sickness is related to the interactive effect of VR mobile terminals [53], with more immersive and interactive VR increasing the likelihood of motion sickness [55]. The vast majority of palliative care patients experience nausea and vomiting, and the prevalence of nausea and vomiting is ≥ 40% in the last 6 weeks of life [56]. Therefore, the interactive design of VR for palliative care patients should find a balance between fun and inducing symptoms of nausea and vomiting, and the addition of interactive content should be performed while maintaining maximum comfort.

Patients are more likely to have an immersive VR experience in a quiet and private environment [30]. Auditory interference may force participants to allocate attentional resources to external and irrelevant stimuli [57], affecting their interaction with the VR environment during therapy. Privacy and dignity were identified as critical for palliative care patients [58]. When privacy and dignity are invaded by an object or even a conspecific, people will generate defensive or avoidance behavior for the sake of self-protection[59], leading to the effectiveness of VR therapy being compromised. Hartigan et al. [60] have concluded that single-occupancy rooms, partitions by walls rather than curtains, gave patients more confidentiality and security during treatment. The presence of others may affect the participant’s interaction with the avatar, The influence may be related to whether or not there is an intimate physical and emotional connection with the patient [58]. Guertin-Lahoud et al. [24] found that participants who had a VR experience with a close companion (friend or partner) had a more positive emotional experience than those who explored alone.

Therefore, during VR therapy, guaranteeing the privacy and confidentiality of the environment and avoiding interruptions by external environmental interference to maintain the continuity of the experience is essential. Another aspect worth considering is inviting really close people to participate in the interactive experience.

#### Advantages of VR therapy: relieving symptoms of discomfort and enhancing self-awareness value

Our study showed that VR therapy has some value in relieving pain in palliative care patients[39, 42, 45], however, more robust data are needed in the future to demonstrate the effectiveness of VR therapy in relieving pain in palliative care patients. VR interventions have been previously shown to be effective in relieving acute pain [14, 15]. The main mechanism underlying the effects of VR therapy may be related to distraction, which is one of the simplest psychotherapies for acute pain, and can help reduce the level of acute pain [61]. The patients were distracted by immersive VR interactions, causing the patient to react more slowly to incoming pain signals and therefore reducing the patient’s perception of pain [16, 17]. VR therapy has also shown its usefulness in reducing mental and psychological burdens [21, 22]. One study [62] monitored the relative gamma power (RG), a biomarker linked to stress, in healthy adults by electroencephalography, and found that participants experienced significant reductions in stress levels during VR use. This connection seems to be related to the landscape design within VR [63]. Stigsdotter’s study [64] pointed out that the impact of natural scenes on positive emotions is positive, e.g., walking in nature alone produced a significant increase in positive emotions. Yeo’s study further emphasized that the natural scenes in VR were equally beneficial for improving subjective well-being [65]. In VR, higher levels of openness, more greenery, more blue skies, and more sunlight exposure yielded higher levels of recovery from perceived depression, anxiety, and stress [63]. Besides, VR landscapes with a higher viewing distance provided more restorative impacts [63].

One of the key takeaways from our research is that patients are able to derive some form of symbolic value from VR therapy. This is an abstract but important finding for palliative care patients. Another interesting phenomenon shown in previous studies [46] is that participants show curiosity about things in the virtual environment, such as pointing at certain elements of the scenery with their hands or “walking” toward these elements, even though they may have limited themselves in real life. Generally, the physical condition caused by terminal illness may limit the patient’s activities to a single room. In such situations, VR offered the patients an opportunity to escape from reality [66]. In our study, patients reminisced about their childhood, redefined themselves, and fulfilled their last wishes through VR. A previous study reported similar results, that is terminal values of older participants gained in VR therapy, including good memories, self-gratification, and sense of belonging [67]. These findings may be related to two aspects of VR therapy, namely, the realism of VR and the use of semi-structured narrative modes in VR therapy. The first aspect is the realism of VR. In some VR storylines, the user needed to complete some tasks for the virtual partner, allowing the participant to abandon the role of a patient and take on the role of an active helper in the process [68]. The “realistic” simulation experience in VR during this process had a positive meaning for patients. Immersive VR can stimulate associations between VR and reality (e.g., reminiscing about the past) [39, 67] and perpetuate the memories and emotions generated in VR to reality [67]. The second aspect is the use of semi-structured narrative modes in VR therapy. We found that patients were more likely to report receiving symbolic value when their interventions included more semi-structured guided VR therapy [39, 41]. In palliative care, patients sometimes fear losing their identity or self-image [7]. The combination of VR and storytelling can combine the advantages of visual guidance and storytelling [11, 69], guiding patients to re-recall and understand their experiences by sharing stories, and rediscover their selves.

#### Mode of VR therapy: coexistence of standardization and personalization

This study showed good acceptance of and different preferences for VR therapy in palliative care [21, 23, 26, 27]. Participants always expressed a need for personalized VR therapy [67]. Personalized VR interventions are associated with better outcomes and experiences for participants than standardized interventions [15]. In a personalized virtual environment, immersion, pleasure, engagement, and relaxation are more likely to be achieved [70]. Personalized avatars significantly increase participants' bodily ownership, presence, and dominance in comparison with regular avatars [71]. These may be related to differences in physical fitness and life experiences between individuals [70], and even to age [67, 72]. For example, older participants are more inclined to prefer fun, safe, and relaxing VR leisure activities, and expect VR to improve their relationships with others, quality of life, and sense of belonging [67]. For palliative care patients, which have different circumstances, personalization is important. However, in comparison with personalized therapy, standardized VR therapy can provide greater reproducibility and productivity and provide a predictable patient experience [73]. Moreover, personalized therapy will increase the workload of the team and the cost of VR therapy [73, 74], highlighting the need to maintain a balance between individualization and standardization. Gregory et al. [75] offers a possibility of personalization, which is to add a modular therapy in the case of standardized therapy, which can be selected according to the specific characteristics of the individual (based on research evidence, as well as patient preferences). Future research should find the sweet spot between standardized and personalized VR treatment modes. Personalized options are provided for participants in a standardized mode, resulting in different permutations and combinations based on the patient's own choice, resulting in a VR session that connects with that patient.

### Strengths

To our knowledge, this is the first systematic review to synthesize the results of qualitative research on the experiences and perceptions of palliative care patients toward VR therapy. The search was accurately reported in accordance with the ENTREQ statement, and thematic analysis methods were used to extract key themes and evidence and enhance the reliability of the data. We also evaluated the consistency of the quality evaluation by the two investigators, which enhanced the reliability of the research process.

### Limitations

This review had some limitations that require acknowledgment. First, the nine studies showed high heterogeneity in a number of factors, such as the equipment used in the VR intervention (e.g., glasses and monitors), the videos viewed, and the mode, environment, timing, and frequency of the VR interventions. In addition, one of the studies involved a minor participant, and the need to distinguish between adult and pediatric patients is a point worth consideration, since the patterns and focus of palliative care in children and adults are different. None of the studies evaluating adult patients grouped the patients by age, and the usage of newer technologies may differ between young and older people. Second, none of the studies reported the expected price of VR therapy, which could have influenced patients' attitudes toward its use. Moreover, most of the included studies did not elaborate the underlying theoretical frameworks.

## Conclusions

This review reported the experiences and perceptions related to the use of VR therapy among palliative care patients. The patients’ feedback covered discomfort caused by VR devices, the positive and negative sense of experience, and the situations that affected the interactive experience. Patients may also be unable to tolerate VR therapy or may show newer forms of discomfort, such as nausea, dizziness, and other manifestations of motion sickness. VR therapy may be an effective approach to relieve patients’ physical pain, relieve psychological emotions, and gain self-awareness value. In addition, patients prefer personalized VR therapy rather than standardized therapy regimens. Participants also reported preferences for the content, timing, and frequency of VR interventions, and future studies should consider these aspects that may help inform future interventional designs and user experience enhancement for VR therapy.

### Supplementary Information

Additional file 1. Search strategy. This document contains detailed search strategy for this review.

Additional file 2. The results of each quality assessment item of the nine articles by the two researchers. This document contains the results of two authors who conducted quality assessments.

Additional file 3. The thematic synthesis process and proofs. This document contains the process of synthesizing the topics of this review, as well as a full introduction.

## Data Availability

No datasets were generated or analysed during the current study.

## Abbreviations

CMT: Compassionate mind training
ESAS: Edmonton Symptom Assessment Scale
MT: Music therapy
UX: User experience
VR: Virtual reality
