# Supplementary material for: Experiences and perceptions of palliative care patients receiving virtual reality therapy: a meta-synthesis of qualitative studies
Source: BMC Palliat Care. 2024 Jul 23;23:182. doi: 10.1186/s12904-024-01520-5 (PMC11267777; doi:10.1186/s12904-024-01520-5)
Supplement: Supplementary file 1 — Additional file 1. Search strategy. This document contains detailed search strategy for this review. [file 12904_2024_1520_MOESM1_ESM.pdf]

Additional file 1. Full database search strategy

Data base	Search strategy
PubMed	("palliative care"[Title/Abstract] OR "end-of-life"[Title/Abstract] OR "end of stage"[Title/Abstract] OR terminal[Title/Abstract] OR hospice[Title/Abstract] OR dying[Title/Abstract]) AND ("Virtual Reality"[Title/Abstract] OR VR[Title/Abstract] OR "Virtual technology"[Title/Abstract] OR "virtual reality goggle"[Title/Abstract])
Web of Science	(TS=("palliative care" OR "end-of-life" OR "end of stage" OR terminal OR hospice OR dying)) AND TS=("Virtual Reality" OR VR OR "Virtual technology" OR "virtual reality goggle")
EBSCO	SU ("palliative care" OR "end-of-life" OR "end of stage" OR terminal OR hospice OR dying) AND SU ("Virtual Reality" OR VR OR "Virtual technology" OR "virtual reality goggle")
OVID Medline	((palliative care* or end-of-life* or end of stage* or terminal or hospice or dying) and (Virtual Reality* or VR or Virtual technology* or virtual reality goggle*)).ab.
Scopus	(TITLE-ABS-KEY ("palliative care" OR "end-of-life" OR "end of stage" OR terminal OR hospice OR dying) AND TITLE-ABS-KEY ("Virtual Reality" OR VR OR "Virtual technology" OR "virtual reality goggle"))
John Wiley	"palliative care" OR "end-of-life" OR "end of stage" OR terminal OR hospice OR dying" in Abstract and ""Virtual Reality" OR VR OR "Virtual technology" OR "virtual reality goggle"" in Abstract
ProQuest	subject("palliative care" OR "end-of-life" OR "end of stage" OR terminal OR hospice OR dying) AND subject("Virtual Reality" OR VR OR "Virtual technology" OR "virtual reality goggle")
知网	主题：（安宁 OR 缓和医疗 OR 临终关怀 OR 安宁疗护 OR 宁养疗护 OR 舒缓医疗） AND 主题：（VR OR 虚拟现实 OR 虚拟现实技术）
万方	主题:(安宁 OR 缓和疗护 OR 姑息关怀 OR 临终关怀 OR 安宁疗护 OR 宁养疗护 OR 舒缓疗护) and 主题:(VR OR 虚拟现实 OR 虚拟现实技术)
Sino Med	#1=("安宁"[常用字段:智能] OR "缓和疗护"[常用字段:智能] OR "姑息关怀"[常用字段:智能] OR "临终关怀"[常用字段:智能] OR "安宁疗护"[常用字段:智能] OR "宁养疗护"[常用字段:智能] OR "舒缓疗护"[常用字段:智能])<br>#2=（"VR"[常用字段:智能] OR "虚拟现实"[常用字段:智能] OR "虚拟现实技术"[常用字段:智能])<br>(#1) AND (#2)
