# Supplementary material for: Experiences and perceptions of palliative care patients receiving virtual reality therapy: a meta-synthesis of qualitative studies
Source: BMC Palliat Care. 2024 Jul 23;23:182. doi: 10.1186/s12904-024-01520-5 (PMC11267777; doi:10.1186/s12904-024-01520-5)
Supplement: Supplementary file 2 — Additional file 2. The results of each quality assessment item of the nine articles by the two researchers. This document contains the results of two authors who conducted quality assessments. [file 12904_2024_1520_MOESM2_ESM.pdf]

Additional file 2. The results of each quality assessment item of the nine articles by the two researchers.

Table 1. Deng.

**Study**	**I1**	**I2**	**I3**	**I4**	**I5**	**I6**	**I7**	**I8**	**I9**	**I10**
Austin	Y	Y	Y	Y	Y	U	U	Y	Y	Y
Kelleher	Y	Y	Y	Y	Y	U	U	Y	Y	Y
O'Gara	Y	Y	Y	Y	Y	U	Y	Y	Y	Y
Ryu	Y	Y	Y	Y	Y	Y	Y	Y	Y	Y
Brungardt	Y	Y	Y	Y	Y	Y	Y	Y	Y	Y
Lloyd	Y	Y	Y	Y	Y	Y	Y	Y	Y	Y
Johnson	Y	Y	Y	N	N	U	U	Y	Y	Y
Ferguson	Y	Y	Y	Y	Y	U	U	Y	Y	Y
Weingarten	Y	Y	Y	U	Y	U	U	Y	U	Y

Table 2. Huang.

**Study**	**I1**	**I2**	**I3**	**I4**	**I5**	**I6**	**I7**	**I8**	**I9**	**I10**
Austin	Y	Y	Y	Y	Y	U	U	Y	Y	Y
Kelleher	Y	Y	Y	Y	Y	U	U	Y	Y	Y
O'Gara	Y	Y	Y	Y	Y	U	U	Y	Y	Y
Ryu	Y	Y	Y	Y	Y	U	Y	Y	Y	Y
Brungardt	Y	Y	Y	Y	Y	Y	U	Y	Y	Y
Lloyd	Y	Y	Y	Y	Y	Y	Y	Y	Y	Y
Johnson	Y	Y	Y	N	N	U	Y	U	Y	Y
Ferguson	Y	Y	Y	Y	Y	U	U	Y	Y	Y
Weingarten	Y	Y	Y	U	Y	U	U	Y	U	Y

Deng * Huang 交叉表

计数

		Huang			总计
		N	U	Y	
Deng	N	2	0	0	2
	U	0	12	1	13
	Y	0	4	71	75
总计		2	16	72	90

对称测量

		值	渐近标准误差[a]	近似 T[b]	渐进显著性
协议测量	Kappa	.819	.078	8.677	.000
有效个案数		90			

a. 未假定原假设。

b. 在假定原假设的情况下使用渐近标准误差。
