# Supplementary material for: Experiences and perceptions of palliative care patients receiving virtual reality therapy: a meta-synthesis of qualitative studies
Source: BMC Palliat Care. 2024 Jul 23;23:182. doi: 10.1186/s12904-024-01520-5 (PMC11267777; doi:10.1186/s12904-024-01520-5)
Supplement: Supplementary file 3 — Additional file 3. The thematic synthesis process and proofs. This document contains the process of synthesizing the topics of this review, as well as a full introduction. [file 12904_2024_1520_MOESM3_ESM.pdf]

Appendix 3. The thematic synthesis process and proofs

**Theme 1: Experiences of palliative care patients in VR therapy.**
**Analytical theme 1: The experience of using VR hardware devices.**
**01-The manner in which the device was to be worn affected the comfort level.**
“Putting on the virtual reality mask over my glasses was uncomfortable. It kind of smashed my frames into my face a little bit.” (55-year-old woman) <Brungardt, 2021><br>PI: “are they still comfortable?”— “Yes they are” at 17 minutes; PI: “still doing okay?”— “Yes I’m fine” at 25 minutes after she mumbled. <Ferguson, 2020><br>Two (17%) participants reported difficulty wearing the HMD comfortably...Two (17%) participants reported sore shoulders attributable to repeated adjustments of the HMD, but the remaining 10 (83%) participants reported no adverse effects. <Johnson, 2020>
**02-VR equipment was heavy.**
Head was too small for the strap; said it was heavy on the nose; required a readjustment. <Ferguson, 2020><br>Took headset off saying it was too heavy—PI asked about any pain from it being heavy—they asked for them to be put on again, but again quickly removed after looking around and saying they were too heavy. <Ferguson, 2020><br>“But the mask is so heavy it pulls” <Ferguson, 2020><br>Some reported headset was slightly heavy (n = 4) <Kelleher, 2022>
**03-No problems were encountered while operating the device.**
Eight (67%) participants stated that it was easy to use, and 7 (58%) stated that they experienced no difficulties during the session. <Johnson, 2020>

Headset was easy to use (n = 19) and comfortable (n = 17); wearing VR headset with eyeglasses was not difficult (n = 3) <Kelleher, 2022>
**04-Operation of the device was difficult.**
Two participants (17%) reported difficulty using a nasal cannula with the HMD, and 2 (17%) reported difficulty learning the button configuration of the remote controller <Johnson, 2020>
**05-Guidance allowed participants to better use the equipment.**
Participants reported initial difficulties using the hand-held device, they could move around the VR environment easily after a few minutes practice.<br>Two participants required instruction over the duration of the study. <Austin, 2022><br>Jordyn thought at first that it was “really weird” to talk to her past-self avatar, but ended up telling some in-depth stories about two pathways to success: the road she traveled, and the road traveled by a close friend which could have been her road; she saw herself “in a different lane” than where she began her life and became teary and unable to see her future self. <Ryu, 2022><br>Kaitlyn who was having trouble connecting with her avatar at first was asked to pretend she was doing sports (“swing a tennis racquet”; “pretend you’re swimming”) which effectively synched her own motion with the motion she saw performed back; this relaxed her and helped her engage in conversation. <Ryu, 2022>
**Analytical theme 2: The experience of the VR therapy process.**
**06-VR therapy was a novel experience.**
Enjoy: “it was different”< Ferguson, 2020>
“I was really depressed but [it was] nice seeing all those places and seeing those different places where you were happy made a big difference.”<Lloyd, 2021>
**07-The process provided a good immersive experience.**

All participants reported high levels of immersion, with most reporting competent control enabling them to see as much virtual nature as possible. <Austin, 2022><br>Although three participants reported virtual features including flora and sky as two-dimensional, all described the virtual environment as being “very realistic”. <Austin, 2022><br>Commented on clouds, houses, and water. “I see blue and the sun; I see lots of lines” <Ferguson, 2020><br>Pointed and commented on crab going into hole—said she saw “prairie dogs” and chuckled—looked down and to the right often at the crab saying: “that they were visiting each other”; “like they’re teasing one another” <Ferguson, 2020><br>“The sounds of the ocean, this is really wonderful, I love the ocean” <Ferguson, 2020><br>“Do you see that down in the hole?”— “now it’s a different time of day, that’s pleasant” <Ferguson, 2020><br>“The sun is coming up... up and down, up and down” <Ferguson, 2020><br>“You could go anywhere, huh? I like the ocean though, this is my favorite.” <Ferguson, 2020><br>Participant 18: “Yes, he did. The fish swimming past.” Interviewer: “Yes, the fish swimming past. So, what was – how would you describe what it was like?” <Lloyd, 2021><br>“Look, I sit down and my hand disappears!” <Ryu, 2022>
**08-The human-computer interaction experience was good.**
“Avatar, you look like I do now!”; “Oh, lucky you... you have hair!”; “well look at you, you’ve got hips there but you still don’t have a butt!” <Ryu, 2022>
“I’m talking to my younger self here but I can only talk about this in the present...

maybe the avatar helps that.” <Ryu, 2022>
**09-The human-computer interaction experience was poor due to monotonous interventional content.**
“Maybe changing the scene with each song and then, like, actually being interactive with what’s going on. it seems kind of boring is what it was.” (25-year-old man) <Brungardt, 2021>
**10-The human-computer interaction experience was poor due to the participant’s environment.**
Only two participants were aware of external noise distractions. < Austin, 2022><br>“I found it very distressful and distracting, when she walked in just then... who was that anyhow... I was in my place and that just blew it all out... if anyone walked in, it would be the same. OK, I’m not ready anymore but let’s go on.” Notably, Sophia and her avatar were no longer able to be “in sync” after that point of the video, and she responded to all remaining prompts flatly without further character development.<br>< Ryu, 2022>
**11-The human-computer interaction was poor due to the presence of others.**
She was not able to apply (and in fact, directly avoided) talking about her own “pre-family” life growing up, “well, I can’t talk about that here.” The instrumental connection was made, but psychologically her life review process was impacted by the physical presence of loved ones, which limited playful engagement with the avatar. < Ryu, 2022>
**12-VR caused some discomfort.**
Two participants reported mild non-limiting nausea with 3D HMD VR, while two other participants reported initial but transient dizziness in the virtual environment.<Austin, 2022>

“My overall reaction was claustrophobia.” (73-year-old man) < Brungardt, 2021>
**13- Tired or frustrated due to VR therapy.**
Answered affirmatively to have the headset removed, “Yes, I’d like to lay down”<br>< Ferguson, 2020><br>“I find breathing exercises really frustrating … I have tumours in my lungs, the amount I can inhale, the amount of time I can hold for is less than for other people. So, someone will say hold it this many beeps and then you can’t … you feel like you failed at it and you check out…” < O'Gara,2022>
**Theme 2: The perceived value that palliative care patients gain in VR therapy.**
**Analytical theme 1: The actual effects of VR therapy in palliative care.**
**14-VR therapy can relieve pain of palliative care patients.**
“All my pain reduced in there.” (56-year-old man) < Brungardt, 2021><br>This capacity to be virtually removed from one’s current physicality allowed some participants to be able to forget their pain. Participant 13: “I had no pain whatsoever. It really took me out of myself.” < Lloyd, 2021><br>Interviewer: “And afterwards you said you didn’t feel pain while you’re having that. Do you remember that feeling?” Participant 19: “Yes, I mean I was thoroughly involved in the um experience which you might say – yes” < Lloyd, 2021>
**15-VR therapy can relieve depression of palliative care patients.**
“It gave us something to do while not being able to leave the room, it was a new experience that was exciting and helped to distract from the depressing situation of being isolated from everyone.” < Weingarten, 2020><br>“I could escape from the new diagnosis for 20 minutes. I gained that peace for 20

minutes.'' (69-year-old woman) < Brungardt, 2021><br>''it distracted me from any pain, and loneliness I was feeling'' < Weingarten, 2020><br>''Usually when I used the virtual reality it was because I was feeling very bored (SIC) and stir crazy, not being able to leave the room. It gave me a chance to feel like I was going somewhere or doing something even though I was stuck in the room. It made me feel more at home'' < Weingarten, 2020>
**16-VR therapy was relaxing and calming.**
All participants described satisfaction related to pleasure, peacefulness, enjoyment, escape, relaxation, and most positively, childhood memories, especially when using 3D HMD VR. < Austin, 2022><br>"A sense of euphoria. A sense of peace. A sense of calming that was not there before." (62-year-old man) < Brungardt, 2021><br>"So heartwarming. It just opened my soul. I mean it felt like everything lifted straight up off my chest. I feel so relaxed and comfortable." (54-year-old woman)<br>< Brungardt, 2021>
"When I had both the headset and the virtual reality mask on, my breathing changed and became slower and more calm and I relaxed." (55-year-old woman) < Brungardt, 2021>
"good" "so comfortable... so soothing" < Ferguson, 2020><br>- "very relaxing. Yeah. Eh calming." -Interviewer: "Calming?"-" Very calming. Yes, like being in a different world." < Ferguson, 2020><br>- "Another world." - "Personally I thought certainly it was relaxing." -Interviewer: "And do you remember how you felt afterwards?" - "I was………Calm." < Ferguson, 2020>

The breathing techniques, I started to employ when I was having a scan even though the scan was very short. I thought that was quite useful for that. I hadn’t really thought of that before but I found it actually quite calming. 017 < O'Gara, 2022>
**17-VR therapy helped the palliative care patients feel happiness.**
“Looks better than what I’ve been looking at”; “hmmm that’s pretty”— chuckling and smiling. < Ferguson, 2020><br>Smiled a few times at 10 minutes. < Ferguson, 2020><br>“Yeah; I don’t know what it was but I just felt happier. Yep.” < Lloyd, 2021><br>“It was a joyful thing and even going to Greece was a deliberately joyful thing.” < Lloyd, 2021><br>-Interviewer: “And are you still feeling happier? - “ Yeah. I just think it’s the memory I have of going there.” < Lloyd, 2021><br>“But this was a dream...normally it’s a nightmare...but it was anything but a nightmare: it was a joy; a pleasure. I was so happy. I was like just leave me here; come back and get me whenever you need to but I don’t want to go.” < Lloyd, 2021><br>-Interviewer: “Did it make you feel anything while you were doing it?” - “Happy.” - Interviewer: “OK” - “Yeah. Like life’s worth living” < Lloyd, 2021>
**Analytical theme 2: The value of the self-awareness provided by VR therapy.**
**18-VR therapy evoked precious and beautiful memories.**
Participant 2, who was aged over 100 years, was the oldest participant in the study. She had been feeling low in mood as she anticipated a move to a care home. Using VR allowed her to access experiences from her life and bring back memories that allowed her connect to the past. This was profoundly moving for her and helped to lift her mood following the session.<br>“I love mountains. I always go up the Highlands every year if I can. I love the

mountains. So I think it brings back memories.” < Lloyd, 2021><br>“I was absolutely thrilled to put my home address in and see the house that I was born to seventy-five years ago. And I can be quite an emotional person and I wondered if it would make me cry. I wonder if it was the way I saw it. I didn’t think I would see it as clearly as I did but I also knew there was a distinct possibility that the area around it might have been changed or even demolished. But it hadn’t. I was thrilled to bits.”<br>“So it was very – it was a lovely memory and I had all sorts of childhood memories.”<br>“And also a memory is of my father sleeping in a deckchair on a Sunday. And I was also sitting in a deckchair shelling peas for lunch. It was just lovely. I wasn’t remotely sad. I was actually very energized by it.” < Lloyd, 2021>
Participant 11 also was able to address her experience of coping with functional loss following her illness by reconnecting with the many positive experiences of her life. Being able to re-visit places highlighted and enhanced positive memories.<br>“And that was actually good because recently there’s been a lot of my life which has been involved in loss, tragically. Em, I’m not talking about death; I’m talking about loss of function so for me the things that I remembered and being with my husband – so it was a good thing to do.” < Lloyd, 2021>
“[my family] always – we loved watching cowboy films when we were wee. We were raised with the cowboys. And it was so nice being so close to knowing that a film that John Wayne made, in the Grand Canyon.”<br>“I really felt like I was with my Dad. In the Grand Canyon waiting to meet John Wayne.” “And it was lovely to have the sense the feeling that your family were quite close.” < Lloyd, 2021><br>“It brought back memories of generally we would go there on the Sunday. We lived about thirty-five miles away, so our Sunday outing would have been there. And my

Mum and Dad would have been asking me to get out of the car, and I was only a small child at the time, but all I wanted to do was read my book, lie on a rug and read my book." < Lloyd, 2021>
**19-VR therapy gave a new meaning to old actions for palliative care patients.**
Ellison, describing a childhood lost, "I'm right with it now, but I was mad about it then." < Ryu, 2022><br>Tyson, "Eventually I took up boxing. I used to fight a lot at school, and that channeled it in a good way." < Ryu, 2022><br>William, "I kind of blew it. I had a girlfriend and we broke up. Now I know it was depression but I struggled a long time after that until I finally figured out who I was and what was happening to me." < Ryu, 2022>
**20-VR therapy helped palliative care patients shape and discover another self.**
Jordyn thought at first that it was "really weird" to talk to her past-self avatar, but ended up telling some in-depth stories about two pathways to success: the road she traveled, and the road traveled by a close friend which could have been her road; she saw herself "in a different lane" than where she began her life and became teary and unable to see her future self. < Ryu, 2022>
Sophia, "Friends are important to me. And in high school, that meant cliques where you were in, or you were out. I didn't see that then, though, because I was in one."<br>< Ryu, 2022>
Jordyn, describing her sense of responsibility: "After my parents separated it was just my mom and she worked a lot, so we learned to take responsibility." < Ryu, 2022>
Jordyn, "I shifted into a different lane once I met my husband; it wasn't just about me anymore." < Ryu, 2022>
Jordyn (tearful): "I just can't see myself old, I just don't think it's possible." She later

says, “its ok, though. It was very therapeutic to say that out loud.” < Ryu, 2022>
**21-VR therapy helped palliative care patients get rid of the shackles of reality to realize their wishes.**
“I can just put on headphones and kind of space out for a while and be in my own little world that I get to pick out. To a place you want to be. Not somewhere you have to be or stuck. Which is a piece of control you can take back a little bit.” (24-year-old woman) < Brungardt, 2021>
Participant 15 had wanted to visit other parts of the world; however, her terminal diagnosis in her early 40s had cut short her capacity to do so.<br>“I’ve always wanted to go [to New York] but obviously I can’t fly, so I won’t be able to go, but um, yeah it was really good” “It was different; it was really good.” < Lloyd, 2021><br>“Just seeing the world, because not everyone can.”<br>“Just being able to see physically the buildings and the surrounding areas and stuff like that. It was really good.” < Lloyd, 2021><br>Participant 9 had been disappointed about missing a planned trip to New Zealand and was able to virtually visit during her session. The surprising element was that she was able to find peace with the idea that she had not really missed out on as much as she had thought she would have. Looking down the city streets and even across some scenic sights had proved underwhelming. < Lloyd, 2021><br>“It kind of proved that I wasn’t really missing anything by not going to New Zealand.” < Lloyd, 2021><br>“Because he [husband] could never ever take me [to all the places that I wanted to see in the world]. You know. Much as he wanted to, he can’t. He’s got a bad heart so he can’t do anything. Anyway so, we just sat and talked about it the whole night.” <

Lloyd, 2021><br>“And then I’ve always been interested in art.”<br>“There were so many flowers and I kept looking and each one you saw was bigger than that one and I would never get to see that amount of flowers in a lifetime. You know. And I’ve always wanted to go to Monet’s garden, just to see the waterlilies. And I did, and I’ve seen them. It was amazing.” < Lloyd, 2021>
**22-VR therapy provided new expectations for palliative care patients.**
“Yeah. Like life’s worth living.” < Lloyd, 2021>
“...it gave me something to do and look forward to.” < Weingarten, 2020>
**Theme 3: Perspectives of palliative care patients toward using VR therapy.**
**Analytical theme 1: Attitudes before using VR therapy.**
**23-Worries about VR therapy before using it.**
“I was worried about maybe having a claustrophobic experience but I had none.” (59-year-old woman) < Brungardt, 2021>
**24-Acceptance of VR therapy before using it.**
“It was really easy for me to pick out a playlist. I automatically started thinking about songs that I liked. So, it was really easy for me to think about if I wanted to space out, what I wanted to feel and listen to. The only hard part about it was just wondering what it was gonna be like.” (24-year-old woman) < Brungardt, 2021><br>“…putting the headset on isn’t really a problem … we’re all going to have to get used to some kind of virtual reality at some point … hadn’t tried it before but it was very interesting.” <O'Gara, 2022><br>“But I’ve also been on some of these yoga type things where you just try and relax

and get into the mood and all that kind of thing…I thought it was quite useful for that…the talking was the same.” <O'Gara, 2022>
**Analytical theme 2: Attitudes after using VR therapy.**
**25-Did not want to leave the world created by VR therapy.**
“Is this all I’m gonna see?”<br>“I’m sleepy, but I don’t want to leave this wonderful place” < Ferguson, 2020><br>“But this was a dream...normally it’s a nightmare...but it was anything but a nightmare: it was a joy; a pleasure. I was so happy. I was like just leave me here; come back and get me whenever you need to but I don’t want to go.” < Lloyd, 2021>
“I mean when I came back, to reality. You come back with a sort of bounce!” <Lloyd, 2021>
**26-Lack of attraction for VR therapy.**
“This was a beautiful place, but I’d like to go home now.” < Ferguson, 2020><br>Said they just didn’t enjoy it.<br>“Just not that interesting to me. Sorry I couldn’t help you.” <Ferguson, 2020><br>“Interesting... nothing I can’t live without.” < Ferguson, 2020>
**27-Not interested in other parts of the process in the VR intervention.**
“…the compassionate mind therapy, I couldn’t see the point of at all … you are in a compassion rich environment … Nurses, the Doctors, friends and family… the last thing you … need is another dose of compassion…” 027 < O'Gara, 2022>
**28-The effect produced by VR therapy persisted beyond the session.**
For participant 15, this feeling of fulfillment extended beyond the session itself to the following day. < Lloyd, 2021>
**29-The improvement effect of a single VR session was short and limited.**
Six (50%) participants reported that they would feel more “involved” with the VR if

they were given multiple sessions. < Johnson, 2020><br>“I don’t think it will have a lasting impact…It definitely made the rest of the day easier … But the next day, the day after, I didn’t still have that same sense of calm, it was just kind of immediately after…” < O'Gara, 2022>
**30-Attitudes toward using VR therapy again.**
Most participants stated they would use VR regularly if available for pain but also for emotional relief and relaxation. < Austin, 2022><br>“A wonderful experience. If I had my way I would do that every day.” (54-year-old woman) < Brungardt, 2021><br>Do Again: “Depends on the program”<br>“It’s a one-time experience, you don’t need it twice”<br>“What else do I get to see?” < Ferguson, 2020><br>The majority of participants seemed optimistic about the potential for VR therapy in the palliative care setting, with 10 (83%) participants stating they would recommend it to a friend going through a similar situation. < Johnson, 2020>
**Analytical theme 3: Preferences for the VR therapy intervention.**
**31-Preferences for the timing of the VR intervention.**
“More towards nighttime. When there’s less going on.” (25-year-old man)<br>< Brungardt, 2021><br>“I don’t know the optimal time. Early mornings, I love. But it seems like that’s when I’m most emotional and upset is really the morning.” (69-year-old woman) <Brungardt, 2021><br>Evening (n = 10); afternoon or evening (n = 6); afternoon only (n = 3); anytime (n =1)<br>< Kelleher, 2022><br>Would use VR Blue at the time of cancer diagnosis (n = 6), in alignment with other

cancer treatments (n = 12), later during cancer experience (n = 4), and/or during times of particularly high pain, anxiety, or depression (n =7) < Kelleher, 2022>
**32-Preferences for the VR intervention frequency.**
Would use VR Blue everyday (n = 6) • Would use VR multiple times per week (n = 11) < Kelleher, 2022>
**33-Tending to a personalized VR intervention length.**
“You could do several different lengths. You could do a 10 minute one. Some people would like an hour.” (47-year-old woman) < Brungardt, 2021>
**34-Using VR independently.**
Most participants felt that partners/caregivers would be interested and willing to be involved in the use of VR Blue (n = 12) < Kelleher, 2022><br>“I prefer to explore it alone.” < Weingarten, 2020>

2
